# Proteome Analysis of the Six-Eyed Sand-Spider *Sicarius thomisoides* Venom

**DOI:** 10.3390/toxins17100486

**Published:** 2025-09-28

**Authors:** Tomás Arán-Sekul, Juan San Francisco, José Rojas, Kyung-Mee Moon, Leonard Foster, Alejandro Catalán

**Affiliations:** 1 Molecular Parasitology Research Laboratory, Department of Medical Technology, Faculty of Health Sciences, University of Antofagasta, Antofagasta 1270300, Chile; tomas.aran@uantof.cl (T.A.-S.); juan.sanfrancisco@uantof.cl (J.S.F.); jose.rojas@uantof.cl (J.R.); 2 Michael Smith Laboratories, Department of Biochemistry and Molecular Biology, University of British Columbia, Vancouver, BC V6T 1Z4, Canada; kyungmee@mail.ubc.ca (K.-M.M.); foster@msl.ubc.ca (L.F.)

**Keywords:** *Sicarius* spider, Sicariidae spider family, phospholipase D toxins, *Sicarius thomisoides*

## Abstract

Spiders of the *Sicarius* genera (Araneae: Sicariidae) are commonly known as six-eyed sand spiders. Of the species described in Latin America, the species *S. thomisoides* has previously been shown to possess venom with a toxic potential comparable to that observed in the venom of the spider *L. laeta*. Although identifying the phospholipase D activity in the venom of *S. thomisoides*, it is still unknown what other components are part of the venom. In this study, we described the identification of the main protein components of *S. thomisoides* venom, revealing that the phospholipase D family were the majority toxins, followed by Astacin-like metalloproteinases and serine proteases. Additionally, the presence of CRISP-type allergens and peptides from the U-PHTX-Pmx family was identified for the first time in venoms from *Sicarius* genera. Identifying the components of the *Sicarius* spider venom is an essential step to understanding its toxicological potential.

## 1. Introduction

The genus of *Sicarius* spiders (Walckenaer, 1847) corresponds to one of the three genera that make up the family Sicariidae, which also includes the genera *Loxosceles* and *Hexophthalma* [1]. This genus of spiders is composed of 21 species of Haplogyne araneomorph spiders with cryptic habits, known for their ability to cover themselves with substrate as a camouflage mechanism, a behavior described as “masking,” and from which they are known as “six-eyed sand spiders” [2]. These spiders are geographically distributed in arid and semi-arid environments of southern South America, with species that exhibit poor distribution and are often restricted to clearly delimited “island” areas of dry biomes embedded in a matrix of humid habitats [3], while in Chile they are specifically distributed from the northern border with Peru in the Atacama Desert to the central regions of Chile [4,5]. *Sicarius thomisoides* is the endemic species that is most widely distributed in this country. This spider has been found both inland and along the coast, particularly in dry riverbeds and ravines. It is distinguished by its hemisynanthropic habitat, where it could be found in both natural and urban areas in similar proportions [6]. It is characterized by having a large body (15–25 mm), simple genitalia, eyes arranged in three pairs, vertically implanted chelicerae, and a marked sexual and behavioral dimorphism, where females tend to remain sedentary once they establish a niche, while males show greater mobility [4,7]. From an evolutionary standpoint, it is a lineage with a Gondwanan origin, whose divergence from its African sister group is estimated to be approximately 95 million years ago [8].

The knowledge about the composition of the venom of spiders from the genus *Sicarius* is limited, unlike what has been reported for the venom of spiders from the genus *Loxosceles*, despite both genera belonging to the same family Sicariidae and sharing certain toxicological characteristics [9]. Various studies have identified the presence of phospholipase D (PLD) enzymes in *Sicarius* spider venoms, which would exhibit structural and functional similarity to those found in the venom of *Loxosceles* and have been demonstrated to have hemolytic, cytotoxic, and dermonecrotic effects [9,10,11,12,13]. Furthermore, the venom of *S. thomisoides* has been found to include a major protein group between 32 and 35 kDa using proteomic techniques like two-dimensional electrophoresis [9]. This mass range is in line with the molecular mass reported for PLDs in *Loxosceles* spiders [14]. Furthermore, Western blot assays demonstrated cross-reactivity between recombinant PLDs from *Loxosceles laeta* and components of the venom from the three Latin American *Sicarius* species studied to date, *S. thomisoides* [9], *S. ornatus* [12], and *S. tropicus* [13], suggesting significant antigenic conservation. However, interspecific variations in toxicity and venom electrophoretic protein profiles within the genus have been documented. Thus, studies have indicated that the sphingomyelinase D (SMaseD) activity of the Central American species *S. rugosa* is lower compared to the venom of *Loxosceles arizonica* as well as compared to the South African species *S. damarensis* and *S. hahni* [15]; however, the latter are now classified within the genus *Hexophthalma* (*H. damarensis* and *H. hahni*) [1]. Despite the above, it was shown that the three species exhibit distinct electrophoretic patterns in one and two dimensions; however, they share protein components ranging from 31 to 32 kDa, which have been extensively linked to the family of PLD toxins (including sphingomyelinase D) found in *Loxosceles* spider venom [15]. Furthermore, in animal models, formerly classified *Sicarius* species, such as *Sicarius albospinosus* (today reclassified as *H. albospinosa)* and *Sicarius testaceus* (today reclassified as *H. hahni*), have been demonstrated to cause severe systemic abnormalities such as disseminated intravascular coagulation, induce necrosis, and cause local hemorrhage [4,16]. Therefore, these differences indicate the existence of functional variability in the venoms of the genus *Sicarius* and within the family Sicariidae, reinforcing the need to characterize the protein components of representative species from the three genera of the Sicariidae family to understand their toxicological potential.

The identification of sphingomyelinase D activity (PLD toxins) in the venoms of different *Sicarius* species has allowed for the extrapolation of the toxicological potential of these venoms, given the toxin-dependent effects reported from the venoms of *Loxosceles* spiders associated with the family of PLD toxins. These toxins catalyze the hydrolysis of sphingomyelin and other lipids, including lysophosphatidylcholine and lysophosphatidylethanolamine, with variations depending on isoforms and species of *Loxosceles* [14,17,18], and its reported toxic effects include complement-dependent hemolysis, cellular cytotoxicity, endothelial damage, platelet aggregation, and skin necrosis [14,19]. The present study described for the first time the proteome characterization from the venom of the spider *S. thomisoides*, comparing the venom profiles through 2D electrophoresis with those of *L. laeta*, identifying the venom fractions that exhibit the SMase D activity, and utilizing mass spectrometry analysis to identify the family of PLD toxins as the principal toxins from the *S. thomisoides* venom.

## 2. Results

### 2.1. Bidimentional Electrophoresis of Sicarius thomisoides Venom

**Figure 1:**
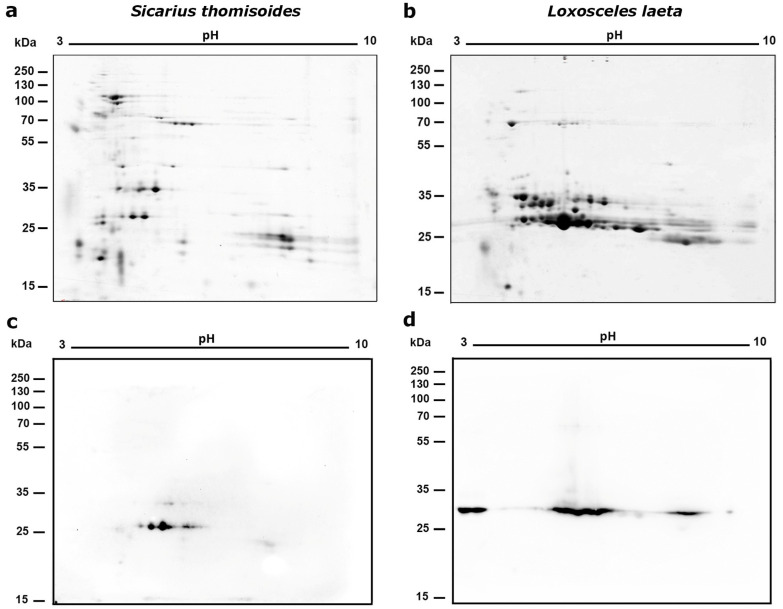
Two-dimensional electrophoresis of *S. thomisoides* venom with an immobilized pH gradient of 3–10. (**a**,**b**): A quantity of 80 μg of total *S. thomisoides* venom proteins was separated in a first dimension using 7 cm isoelectric focusing (IEF) strips with an immobilized pH gradient of 3–10. This was followed by a second dimension using SDS-PAGE separation on a 15% polyacrylamide gel and then stained using SYPRO Ruby staining. Additionally, venom from *L. laeta* was separated for comparison. (**a**) 2D electrophoresis of *S. thomisoides* venom. (**b**) 2D electrophoresis of *L. laeta* venom. (**c**,**d**): Western blot for the detection of phospholipase D. Then, the proteins from the gels for the venoms of *S. thomisoides* and *L. laeta* were transferred to a nitrocellulose membrane, blocked with PBS-Tween20 0.1% + BSA 2%/milk 3% overnight, followed by incubation with polyclonal mouse anti-rLlPLD1 serum (1:1000 dilution). The reaction was detected by incubation with a 1:40,000 diluted mouse anti-IgG (H+L)-HRP conjugate antibody and revealed using electrochemiluminescence (ECL). (**c**) *S. thomisoides* venom. (**d**) *L. laeta* venom.

The *S. thomisoides* venom was separated by two-dimensional electrophoresis (2D-PAGE). It was found that the venom has protein spots with pI values between 3 and 9, mostly clustered in the molecular mass located in the 20–35 kDa range. One of them, below 20 kDa and with acidic pI (~3–4), a second group below 20 kDa but with pI ~8–9, a third group of protein spots between 25 and 32 kDa (pI ~3.9–5.5), another group of spots located at 35 kDa, and a last group of spots between 55 and 100 kDa mainly belonging to an acidic pI (~3.2–5.5) were present (Figure 1). On the other hand, when comparing the protein spot profile with the 2D electrophoresis profile of *L. laeta* venom, the concentrated spots between 25 and 35 kDa were mainly associated with a more neutral pH, and there were not many spots in the 70 kDa range (Figure 1b). Along with this, it was evident that the two venoms of *S. thomisoides* and *L. laeta* differed in the number of protein spots, with the latter containing more (Figure 1c). Furthermore, the Western blot using a polyclonal serum against the recombinant PLD1 protein of *L. laeta* was able to detect three to five protein spots between the 25–35 kDa range in the *S. thomisoides* venom (Figure 1b), similar to protein spots detected in the venom of *L. laeta* at the same 25–35 kDa mass range, which is typical for these enzymes (Figure 1d). Given that phospholipase D toxins are known at 25–35 kDa in *L. laeta*, it is possible that the proteins observed at 30 kDa in the *S. thomisoides* data may also be phospholipase D toxins.

### 2.2. Fractionation of the Venom of Sicarius thomisoides by RP-HPLC

**Figure 2:**
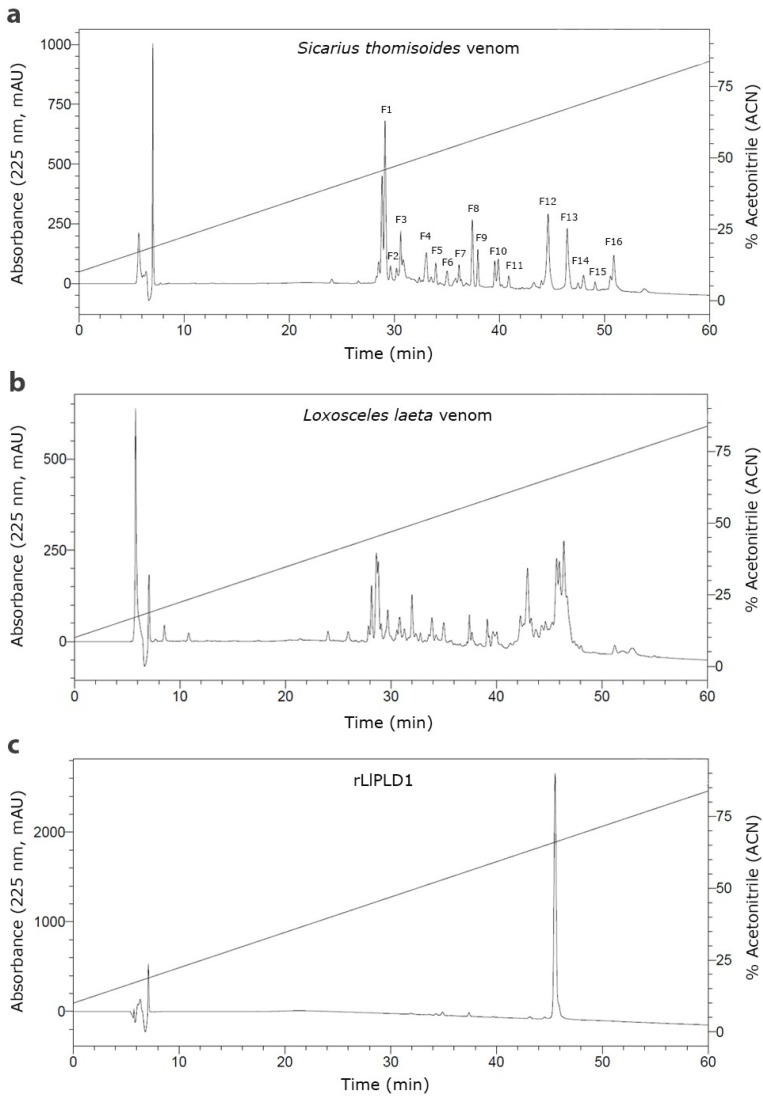
Comparative reverse-phase high-performance liquid chromatography (RP-HPLC) of the venom of *S. thomisoides*, *L. laeta*, and the recombinant protein rLlPLD1. RP-HPLC was performed on 40 μg of protein from *S. thomisoides* venom (**a**), *L. laeta* venom (**b**), and recombinant rLlPLD1 protein (**c**), using a Viva C18 semi-preparative reversed-phase column (250 × 4.6 mm), 5 μm, 300 Å (Restek Corporation), with a linear gradient of 10 to 90% acetonitrile (ACN) in water + 0.1% TFA over 60 min at 25 °C, at a mobile phase flow rate of 0.5 mL/min. The absorbance peaks at 225 nm were expressed as mAU (left Y-axis) and plotted against the assay time (X-axis) and the percentage of acetonitrile (right Y-axis).

**Figure 3:**
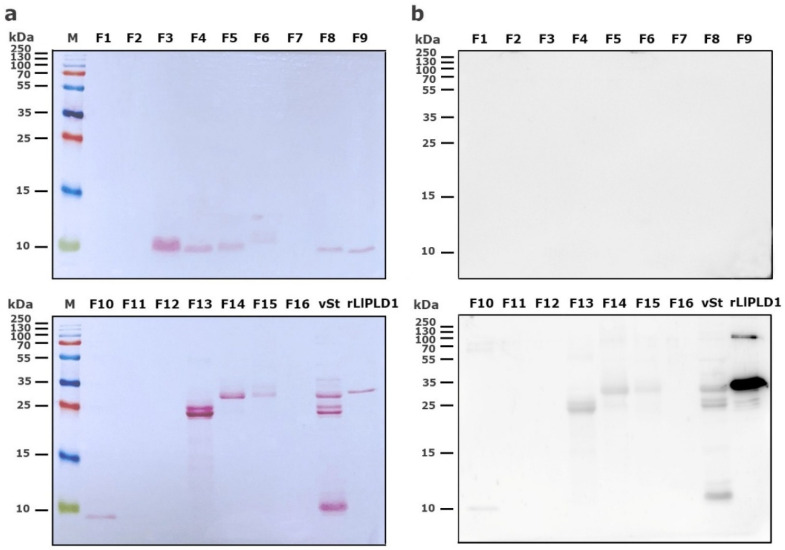
Detection of HPLC fractions of *S. thomisoides* venom using Western blot with polyclonal anti-PLD antibodies. (**a**) The venom of *S. thomisoides* (100 μg) was fractionated by RP-HPLC, and the fractions (F1 to F16) were recovered and resuspended in 20 μL of 1X protein loading buffer. The fractions were separated by SDS-PAGE electrophoresis on a 15% gel and visualized with Ponceau red staining. (**b**) The fractions were transferred to a nitrocellulose membrane and blocked with 2% BSA/3% non-fat milk in PBS-Tween 0.1% overnight, followed by incubation with a pool of polyclonal antibodies against rPLD diluted 1:1000 in PBS-T buffer. Detection was performed with goat anti-mouse IgG (H+L) antibody conjugated with HRP at a 1:40,000 dilution in PBS-T, followed by incubation with ECL reagent. vSt: *S. thomisoides* venom control (5 μg); rLlPLD1: recombinant protein rLlPLD1 control (2 μg).

**Figure 4:**
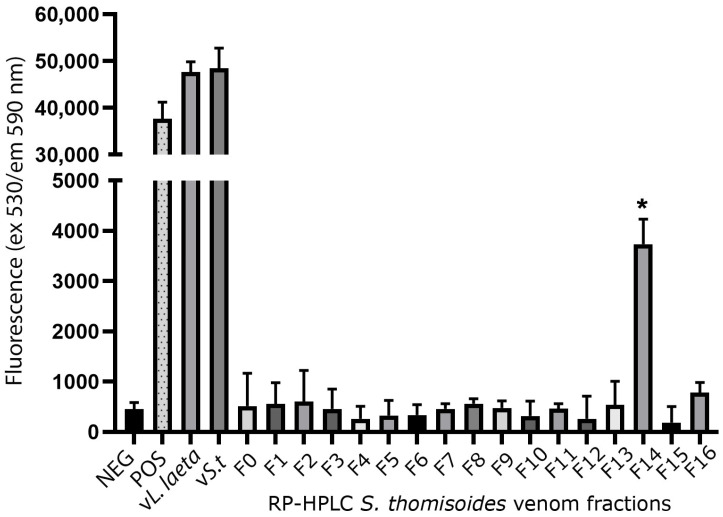
Phospholipase D activity against sphingomyelin in fractions of *S. thomisoides* venom obtained by HPLC chromatography. The fractions of *S. thomisoides* venom obtained by RP-HPLC were collected and resuspended in 100 μL of Amplex Red reaction buffer and then incubated for 90 min at 37 °C with the Amplex Red reagent of the Amplex Red Sphingomyelinase D Assay. Once the incubation time was complete, fluorescence was read at excitation 530 nm/emission 590 nm. Reaction buffer was used as a negative control (NEG), while H_2_O_2_ was used as a positive control (POS), along with 20 μg/mL of *S. thomisoides* (vS.t) and *L. laeta* (vL.laeta) venoms. The tests were performed in triplicate. * Statistical significance *p* < 0.05. One-way ANOVA with Dunnett’s post-test for mean fraction F14 vs. negative control (*p*-value < 0.0270).

Reverse-phase high-performance liquid chromatography (RP-HPLC) was then used to separate the protein fractions of *S. thomisoides* venom, and the resulting fractions were evaluated to determine which of them are detected by the anti-rLlPLD1 polyclonal serum. In this way, as shown in Figure 2, the chromatographic profile of *S. thomisoides* venom shows at least 16 peaks that are visualized between retention times of 24 and 54 min (Figure 2a); likewise, the chromatographic profile of *L. laeta* venom also shows at least 16 peaks, distributed between the same retention times of 24 and 54 min (Figure 2b). Also, this retention time for the fractions of *S. thomisoides* venom was compared with the chromatographic profile of the recombinant protein rLlPLD1 (Figure 2c), whose retention time is shown as a single peak close to 45 min of retention. Additionally, the protein fractions F13, F14, and F15 collected between retention times of 45 to 50 min and with a molecular mass between 25 and 35 kDa from the venom of *S. thomisoides* were recognized by the anti-rLlPLD1 polyclonal serum, just as well as the complete venom of *S. thomisoides* or the positive control with the rLlPLD1 protein, confirming that these fractions contain phospholipases D (Figure 3). Along with this, and to corroborate whether the collected fractions F13, F14, and F15 correspond to phospholipases D, the activity against sphingomyelin was evaluated for each of the fractions, where it was possible to observe that fraction F14 exhibited PLD activity against sphingomyelin significantly compared to the negative control (reaction buffer) and above the initial fractions (F0, F1, and F2), which were not detected by the anti-rLlPLD1 polyclonal serum (Figure 4).

### 2.3. Identification of Protein Components of S. thomisoides Venom Using Mass Spectrometry

**Figure 5:**
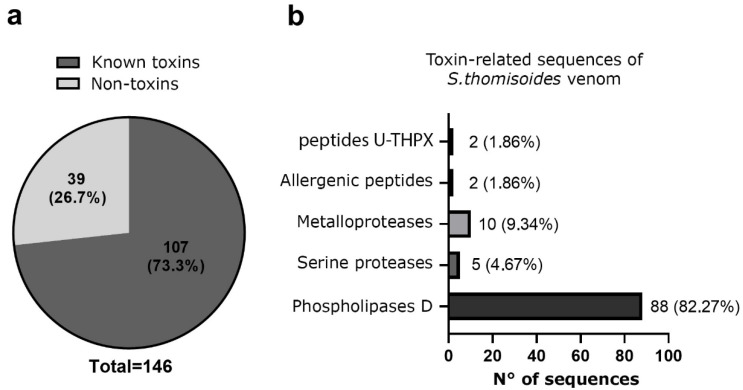
Proteomic analysis of the identified peptide sequences in *S. thomisoides* venom obtained using reverse-phase nano-liquid chromatography coupled with mass spectrometry. (**a**) Pie chart of the frequency of unique peptide sequences identified by LC-MS/MS of *S. thomisoides* venom. (**b**) Frequency of unique peptide sequences for toxins of the venom of the spider *S. thomisoides* identified as toxin-related function.

**Figure 6:**
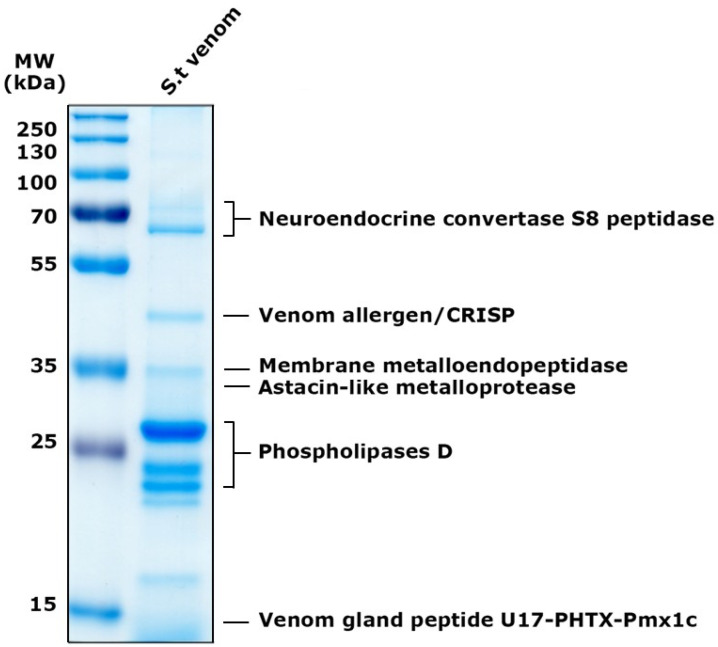
Electrophoretic distribution of proteins from *S. thomisoides* venom identified by MS/MS. SDS-PAGE on a 12% gel for a quantity of 5 μg of *S. thomisoides* venom and stained with Coomassie blue. MW: PageRuler Prestained Plus molecular weight marker from 10 to 250 kDa. St. venom: *S. thomisoides* venom. Proteins identified by MS are indicated on the right side of the gel.

The total venom pool from *S. thomisoides* was submitted to mass spectrometry, resulting in a total of 146 amino acid sequences for unique peptides. These were compared through BLASTp with sequences available in the UniProt database for species of the genus *Sicarius* and the genus *Loxosceles*, allowing the identification of 39 (26.7%) sequences cataloged as non-toxin associated sequences, and 107 (73.3%) sequences were identified as toxin-associated sequences (Figure 5a). The analysis of the venom sequences from *S. thomisoides* showed that 82.27% are toxins from the PLD family, 4.67% are serine protease-type toxins, especially neuroendocrine convertase S8 peptidases, 9.34% are metalloproteases (including membrane metalloendopeptidases and Astacin-like metalloproteases), 1.86% are allergenic CRISP-type venom toxins, and 1.86% are sequences with unknown functions, including the venom gland peptide U17-PHTX-Pmx1c (Figure 5b). From each toxin family it was possible to identify nine sequences of PLDs, five Astacin-like metalloproteases, five neuroendocrine convertase S8 peptidases, two venom gland peptide U17-PHTX-Pmx1c, and two venom allergen/CRISPs (Table 1). The molecular masses of the protein components in the venom of *S. thomisoides* were estimated based on their identified homologs. Thus, the main proteins include PLD toxins ranging from 22 to 30 kDa, astacin-like metalloprotease toxins at approximately 30 kDa, metalloendopeptidases at 34 kDa, CRISP-type venom allergens at 46 kDa, membrane metalloendopeptidases at 59 kDa, neuroendocrine covertase peptidase at 67 kDa, and U17-PHTX-Pmx1c peptides at 10 kDa (Figure 6).

## 3. Discussion

The toxic potential of the *Sicarius thomisoides* spider venom has been previously reported, where the activity of phospholipases D in the venom and its ability to cause complement-mediated hemolysis of red blood cells have been identified, as well as its cytotoxic and dermonecrotic capabilities in a similar manner to that reported for spiders of the genus *Loxosceles* (family Sicariidae) [9]. Furthermore, the *S. thomisoides* spider has been shown to have cytotoxic and dermonecrotic properties similarly to what has been documented for other Latin American species of the genus, including *S. tropicus* [13] and *S. ornatus* [12]. Furthermore, among all the species of the genus *Sicarius* found in Chile [4,20], *S. thomisoides* has been described as the largest and most aggressive; it can even feed on tiny vertebrates or creatures of a comparable size [21].

As arachnids belonging to the Sicariidae family, *S. thomisoides* and *L. laeta* exhibit a broad spectrum of prey in their diet, but with clear differences considering the intradomiciliary environment of the latter, which could influence the differences in the protein components present in each venom [22], as observed in the protein spot profiles of both venoms in 2D electrophoresis. Thus, the differences in the number of protein spots would be attributed to inter-genera variations and can be seen as representative of the protein content of both arachnids in their natural niche environments. This is because the venoms of *S. thomisoides*, and *L. laeta*, used for protein characterization were collected from freshly captured specimens without manipulation or additional feeding in the laboratory during their captivity. This indicates that the protein amount and complexity of the venom produced by the spider in its native habitat were accurately reflected in the recovered venom. Furthermore, since the observed protein profiles correspond to a pool of venoms from all the captured specimens, including nymphal and adult stages (males and females) of each of the analyzed arachnids, they did not include the intraspecies variations that are typically seen according to the biological stage or sex.

These intra-species variations have been previously reported for the protein profiles of nymphs and adult stages of the three species *S. thomisoides* [9], *S. ornatus* [12], and *S. tropicus* [13], with the latter species showing significant differences in venom protein content between males and females, with females having a higher content. Also, spiders of the genus *Loxosceles*, including the species *L. laeta*, exhibit these intraspecific differences in their venoms [13]. Despite the differences shown here between the two venoms, significant similarities could also be seen, mainly in the electrophoretic protein region between 25 and 35 kDa, where the recombinant anti-phospholipase D polyclonal serum from *Loxosceles* was able to detect protein spots in the venom of *S. thomisoides* that were also present in the venom of *Loxosceles* spiders (Figure 1c). This molecular mass range has been reported in *Loxosceles* spiders as containing proteins belonging to the phospholipase D enzyme family, whose presence has been documented in all *Loxosceles* species [11]. Additionally, the presence of multiple paralogous forms of SMase D in diverse *Sicarius* species from Africa and South America has been reported previously, and the estimated molecular weights of expressed proteins are between 31.2 and 32.9 kDa, and the predicted p*I*s of all known *Sicarius* SMase D paralogs range from 5.1 to 9.5, where there is a dense band on one- and two-dimensional gels [10].

The cross-reactivity previously reported by a polyclonal anti-PLD serum from *Loxosceles* has been previously reported by our group in one-dimensional SDS-PAGE [9], along with the observed cross-reactivity between the venoms of *Sicarius* and *L. laeta* using sera from individuals with and without a history of loxoscelism [23], corroborates the similarities between both venoms at this protein molecular mass range. Furthermore, it was possible to identify at least 5 protein spots between 25 and 35 kDa (the molecular weight range for PLDs), indicating a protein family of intra-species toxins (Figure 1). Additionally, in studies conducted with the venoms of the Brazilian species *S. ornatus* and *S. tropicus*, cross-recognition was also observed between a polyclonal serum produced against recombinant SMases D from *Loxosceles* and the venom of male and female *S. ornatus* and *S. tropicus* [12]. The venoms of spiders from the genera *Loxosceles* and *Sicarius* studied to date maintain a significant level of antigenic similarity that allows the cross-recognition and confirms the presence of PLD toxins in the venoms of the three *Sicarius* species studied in Latin America, despite the significant antigenic variations observed intra- and inter-species in isoforms of the PLD family within the genus *Loxosceles* previously reported [24]. In this way, when the percentage of identity between the PLD sequence of *Sicarius terrosus* (syn. *S. thomisoides*) (protein access No. AJV88488.1) [18] and the amino acid sequence of the recombinant protein rLlPLD1 of *L. laeta* (protein access No. ADP00408.1) [25] was compared, both exhibit a 49% identity (data not shown), which could be considered significant, since the identity of PLD sequences within the genus *Loxosceles* ranges from 41.7% to 91.3% in a phylogenetic cluster of less related species [24], thus not ruling out the presence of common antigenic epitopes in the PLDs of both genera.

The venom of *S. thomisoides* was fractionated using RP-HPLC to determine which protein fraction between 25 and 35 kDa would exhibit PLD activity against the sphingomyelin substrate (SMase D activity). Thus, only fraction F14 demonstrated significant PLD activity, in contrast to other fractions detected by the anti-PLD polyclonal serum from *Loxosceles*, such as fractions F13 and F15. The above information would indicate that the proteins with PLD activity against the sphingomyelin substrate correspond to those with a molecular mass of 30–32 kDa; nonetheless, the F15 fraction, which lacked PLD activity, also has the same molecular mass. Therefore, the proteins contained in fraction F15 could correspond to isoforms of the toxin without catalytic activity on sphingomyelin, as has been reported for the PLDs present in the venom of different species of *Loxosceles* spiders [25]. Moreover, it has been reported from the three-dimensional structure of *Sicarius* PLD that these toxins belong to Class II (with the presence of two disulfide bridges) [18,26] and not to Class I (with a single disulfide bridge) [27], which would affect their catalytic activity, as has been reported for *Loxosceles* spider PLDs [28]. In the case of the F13 fraction, which was also identified as PLD by the anti-PLD serum from *Loxosceles* (Figure 3), the explanation could be related to the presence of PLD isoforms with a substrate preference different from sphingomyelin and lysophosphatidylcholine, as has been reported for *Sicarius* PLDs, which show a strong preference for lipids with an ethanolamine headgroup over choline, unlike *Loxosceles* PLDs, since sphingolipids containing an ethanolamine headgroup are common in insect prey [18].

Followed, since there are no proteome studies available for spiders of the genus *Sicarius*, the logical point of comparison for identifying the protein components corresponds to the venom proteomes of spider species from the genus *Loxosceles*. For the reasons mentioned above, databases for the *Loxosceles* taxon were also employed to identify the venom peptides of *S. thomisoides* that were collected using mass spectrometry. Thus, proteins between 31 and 35 kDa with pI between 4 and 10 are primarily found in proteome studies for various species of *Loxosceles*. These proteins have been identified as sphingomyelinases D in the species *L. gaucho*, *L. laeta*, *L. intermedia*, *L. arizonica*, *L. apachea*, *L. deserta*, *L. reclusa*, *L. adelaida*, and *L. similis* [29,30,31,32,33], and they are also shown to constitute an intra-species and inter-species family [11].

Regarding the proteomes described for *Loxosceles* spiders, a report performed in the species *L. intermedia*, identified 39 proteins, of which 10 corresponded to unique proteins in the venom of *Loxosceles*, and were grouped by function into 14 proteins identified as toxins responsible for damage, including aminopeptidases, serine proteases, metalloproteinases, hyaluronidases, phospholipases A2-like, lipase-like, and sphingomyelinases D; another group formed by 15 proteins involved in mechanisms that produce envenomation and proteins as part of strategies for the integrity of the toxins, including serine protease inhibitor, cysteine protease inhibitor, trypsin inhibitor, allergen-like proteins, and the chemotaxis response regulatory protein (CRRP); and a group of 10 housekeeping proteins from the venom glands [34]. In another study also conducted with the venom of *L. intermedia*, the presence of 190 identified proteins was reported, including the group of venom toxins including PLDs, astacin-type metalloproteases, and ICK peptides as toxins with high abundance, as well as others considered in low abundance such as serine proteases, venom allergens, hyaluronidases, and TCTP, in addition to a group of proteins associated with cellular processes [35]. These findings partially correlate with the protein sequences identified through transcriptome studies, since it has been reported in the venom of *L. intermedia* that the main transcripts encoding toxins were not the PLD family but the Knottin proteins (56%), followed by astacin-like metalloproteinases (23%), PLDs (20%), and 1% for other toxins [36]; while in the species *L. laeta*, the transcripts reported for PLDs were only 16% [37]. However, in the same species found in Peru, it has been reported that the percentage of transcripts associated with PLD was the highest found, with 69.28%, followed by metalloproteases (20.72%), sicaritoxins (6.03%), serine proteases (2.28%), hyaluronidases (1.80%), and transcriptionally controlled tumor protein TCTP (0.56%) [38]. Also, the analysis of transcripts from the venom of the species *L. similis* has shown the presence of 23 complete sequences for PLD corresponding to 15% of the venom gland transcripts [39]. Along with this, it has recently been reported that the main abundance of transcripts in the venom of the three main species, *L. intermedia*, *L. gaucho*, and *L. laeta*, was predominantly for PLDs, with 65.4%, 71.8%, and 50.4%, respectively. Additionally, the existence of a group of highly expressed toxins, including PLD, metalloproteinases, and ICK peptides, and a second group of less expressed toxins that include serine proteases, serine protease inhibitors, hyaluronidases, TCTP, and neurotoxins, was confirmed in the three species [40].

In the case of the species *S. thomisoides* reported here, the proteome analysis confirms that the major toxins in the venom correspond to the family of phospholipases D (82.2%), with the identification of at least 9 different isoforms, followed by astacin-like metalloproteases (9.34%) and serine proteases (4.67%), which is consistent with the proteins reported for spiders of the genus *Loxosceles*. Additionally, given that the presence of the PLD family in the venoms of South African species like *Sicarius hahni* and *Sicarius testaceus* (now classified as *Hexophtalma hahni*) had also previously been reported, the latter suggests a protein conservation of the venom components for the three genera of the Sicariidae family, particularly in their major components [41].

The significance of the PLD toxin family has been extensively established, since it has been reported to be the main toxic component found in the venom of spiders belonging to the genus *Loxosceles* [14]. Furthermore, the different recombinant isoforms of PLD that exhibit sphingomyelinase activity can recreate the main symptoms of loxoscelism, including hemolysis, platelet aggregation, nephrotoxicity, and dermonecrosis [42]. The detection of at least 5 protein spots in the venom of *S. thomisoides* by the anti-PLD polyclonal serum from *Loxosceles* and the subsequent identification of a group of at least 9 different isoforms by MS (Table 1) confirms the presence of an intra-species family of toxins, as has been reported in the venom of *Loxosceles* spiders. On the other hand, the *Loxosceles* astacin-like metalloproteases (LALP) were initially described in the venom of *L. intermedia* [43]; however, they are present in the venom of all species of the genus *Loxosceles* [44,45], as well as extensively in other organisms [46]. In *L. laeta*, nine possible LALPs have been reported [47], and they have been reported to be 8.2% of the transcripts present in the venom [37], while in *L. intermedia*, they correspond to 9.8% of the transcripts [36]. These toxins are extracellular zinc-dependent metallopeptidases and can be considered the second most relevant toxic component in *Loxosceles* venom, as they exhibit proteolytic activity on various extracellular matrix protein components, such as gelatin, fibronectin, fibrinogen, and entactin, and are associated with the dissemination of other toxins, as well as the hemostatic effects [48,49,50]. Therefore, being the second most identified protein component in the venom proteome of *S. thomisoides*, with at least five possible isoforms (Table 1), it seems necessary to investigate whether the family of astacin-like metalloproteinases in the venom of this spider can generate the same effects observed for astacin metalloproteinases reported in the venom of *Loxosceles*.

Additionally, serine proteases of the neuroendocrine convertase S8 peptidase type were identified in the venom of *S. thomisoides*. The serine proteases have been reported in the venom of *L. intermedia* with molecular masses ranging from 85 to 95 kDa and ideal pH values between 7 and 8 [51]. Furthermore, these enzymes have been identified as being present in 0.5% of the transcripts in the venom of *L. laeta* [37] and 0.3% of the transcripts in the venom of *L. intermedia* [36]. About the serine proteases like neuroendocrine convertase S8 peptidase, these correspond to convertase enzymes whose members include furins and kexins, also known as subtilases [52], and have been reported in the venoms of spiders such as *Physocyclus mexicanus* [53]. The function of these enzymes in the venom of *S. thomisoides* may involve activating other components present in the venom through precursor cleavage and serving as digestive enzymes to break down proteins found in prey insects [49], similarly to the reported cleavage of latrotoxin precursors in *Latrodectus* spp. spiders [54]. Moreover, the peptides found in the *P. mexicanus* venom, including the U-PHTX family, contain recognition sites for furin, suggesting that their functional maturation could also depend on the action of these serine proteases [53].

Regarding the U-PHTX peptides in the venom of the *S. thomisoides* spider, we were able to identify at least two peptides homologous to U17-PHTX-Pmx1c from *P. mexicanus*. In this venom, proteomic and transcriptomic analysis [53] identified 17 peptides from the family named U1-U17 PHTX-Pmx, with molecular masses ranging from 3.6 to 7.8 kDa. The peptides identified were mostly homologous to venom peptides that possess an Inhibitor Cysteine Knot (ICK) fold, except for peptides U16 and U17 [53]. Thus, the two peptides identified in *S. thomisoides* showed a 100% sequence identity with the peptide U17-PHTX-Pmx1c from *P. mexicanus* (data not shown), which means they would not correspond to an ICK peptide. However, the ICK peptides have indeed been identified as one of the major components in the transcriptomes of *L. intermedia* [55,56]. These ICK peptides, also known as knottins, are characterized by their neurotoxic properties, as they act on ion channels and receptors expressed in the nervous system of insects and mammals, and their effect could be associated with paralyzing and killing prey and predators [57]. However, unlike what is observed in *Loxosceles* venoms, where ICK peptides are noted as one of the main components of the venom [36], their presence in the venom of *S. thomisoides* seems to be a minority. Despite that, two peptides called U1-sicaritoxin-Sd1a and U2-sicaritoxin-Sd1a, which contain the ICK peptide motif, have been reported in the venom of *Sicarius dolichocephalus* [58].

Additionally, it was possible to identify CRISP venom allergen proteins in the venom of *S. thomisoides*. These proteins, known as Cysteine-rich secretory proteins (CRISPs), are a protein superfamily found in a wide range of organisms, from the mammalian male reproductive tract to the venom of lizards and snakes [59,60,61]. The CRISP superfamily includes proteins such as Allergen 5 from vespid wasps, Allergen 3 from fire ants, mammalian testis-specific protein (Tpx-1), and pathogenesis-related protein-1 (PR-1) [62,63,64] that have a structure of α-β-α fold, with a common secondary structure that includes 16 conserved cysteine residues. CRISPs also have two main domains, a CAP/PR-1 domain at the N-terminus and a cysteine-rich (CRD)/ion channel regulatory (ICR) domain at the C-terminus, connected by a hinge region [61].

## 4. Conclusions

Here, we report the first study of the proteome of the six-eyed sand spider *S. thomisoides*, demonstrating that the main protein component of the venom corresponds to the family of phospholipase D toxins, which may also exhibit different preferences for lipid substrates. Additionally, it was possible to identify toxins in the venom frequently found in *Loxosceles* spider venoms, such as Astacin-type metalloproteinases and serine proteases. Finally, the presence of CRISP allergens in the venom of *Sicarius* spiders is reported for the first time, as well as homologous U-PHTX-Pmx peptides that lack ICK motifs. The description of the toxic components of *S. thomisoides* venom will allow for a deeper understanding of the potential toxic capacity of the venom of this spider belonging to the Sicariidae family and serve as a starting point for future studies evaluating the function of each protein component.

## 5. Materials and Methods

### 5.1. Spiders and Venoms

Twenty-five nymphs and adults of *S. thomisoides* spiders were captured in desert and semidesert areas of the La Chimba National Park in the Antofagasta Region, located in northern Chile. Additionally, twenty *L. laeta* spiders were captured from domiciliary habitats in the city of Antofagasta, Chile. All the spiders were maintained at the Molecular Parasitology Research Laboratory at the University of Antofagasta, Chile. The *L. laeta* specie was identified using the morphological characteristics reported by Gertsch (1967) [65]; the specimens were then classified according to stages in adults (males and females) and nymphs. While *S. thomisoides* spiders were identified using morphological characteristics reported by Magalhaes et al. (2017) [4], the specimens were classified according to stages in adults or nymphs.

*S. thomisoides* venom (vSt) and *L. laeta* venom (vLl) were extracted by electrostimulation from the spiders after a week of captivity and with no previous feeding during captivity. Then, the venom droplets were collected with a micropipette in 30 μL of PBS, pooled, and stored at −80 °C until use, as previously reported [9]. The protein concentration of venom samples was evaluated using the Pierce™ 660 nm Protein Assay kit (Thermo Scientific Inc, Rockford, IL, USA), and the protein concentration in μg/mL was calculated from a standard BSA curve ranging from 125 to 2000 μg/mL. All the protocols for the biological research in invertebrate and biotechnological species, including the procedures for spider capture, spider maintenance, and venom extraction, were approved by the Ethics Committee in Scientific Research of the University of Antofagasta (CEIC-UA) (code: CEIC-REV No. 06/2019, date: 12 June 2019).

### 5.2. 1D-Electrophoretic Separation of Sicarius thomisoides Venom

The protein components of *S. thomisoides* venom were electrophoretically separated using 12% SDS-PAGE gel. For this, an amount of 10 µg of *S. thomisoides* venom pool was mixed with 6x protein loading buffer and run at a constant voltage of 110 V for 90 min in Tris-Glycine buffer (25 mM Tris, 192 mM glycine, 0.1% SDS, pH 8.3) using a Bio-Rad Mini-Protean Tetra chamber (Bio-Rad, Hercules, CA, USA). The protein bands were compared with a commercial standard of molecular weight between 10 and 250 kDa PageRuler Plus Prestained Protein Ladder (Catalog Number: 26619, Thermo Fisher Scientific, Rockford, IL, USA) and stained with Coomassie Brilliant Blue G-250 (Sigma-Aldrich Co. LLC, Saint Louis, MO, USA), then destained with distilled water overnight for subsequent photo documentation and analysis using the Chemidoc™ Touch gel imaging system (Bio-Rad Hercules, CA, USA).

### 5.3. Bidimentional Electrophoretical Separation of S. thomisoides Venom

An amount of 80 µg of the *S. thomisoides* venom pool was precipitated overnight at −20 °C using an 8:1 mixture of cold acetone and Trichloroacetic Acid (TCA). Then, the sample was washed 3 times with 500 µL of acetone followed by centrifugation at 15,000× *g* for 15 min at 4 °C. Subsequently, the sample was dried using the Speed Vacuum system (Vacufuge plus, Eppendorf, Framingham, MA, USA) to remove all remaining acetone from the washing process. Next, the dry sediment was resuspended in 150 µL of C1 buffer (8 M urea, 1 M thiourea, 4% CHAPS, and 66 mM DTT), with the addition of 1% Destreak solution (GE Healthcare, Buckinghamshire, UK) and 0.8% ampholytes pH 3–10 (Bio-Rad, Hercules, CA, USA). The same amount of protein from a pool of *L. laeta* venom was subjected to the same procedure and subsequently separated. Followed by the first electrophoretic dimension, which was performed using 7 cm isoelectric focusing (IEF) strips with immobilized pH gradient 3–10 NL (Readystrip™ IPG Strip Bio-Rad, Hercules, CA, USA). Then, each strip was rehydrated with the volume of proteins from each venom previously precipitated in C1 buffer for 12 h at 50 V in the Protean IEF Cell system (Bio-Rad, Hercules, CA, USA). To avoid the effect of heating the strip, 1 mL of mineral oil was added to it before starting this process. Then, for the IEF, a 3-stage program was used: 100 V for one hour, 500 V for one hour, and finally 4000 V until a total of 10,000 V per hour was reached. Finally, the proteins separated according to their isoelectric point (pI) were subsequently denatured and equilibrated in two steps: a first reduction step with 2 mL of equilibration buffer 1 (1.5 M Tris-HCl pH 8.8, 6 M urea, 34% glycerol, 2% SDS, and 66 mM DTT) for 15 min and a second alkylation step with 2 mL of equilibration buffer 2 (1.5 M Tris-HCl pH 8.8, 6 M urea, 34% glycerol, 2% SDS, and 230 mM iodoacetamide) for 15 min, with constant agitation. Once this process was completed, the strip was ready for the second-dimension run. Next, the strips were subjected to the second dimension, through electrophoresis under denaturing conditions (SDS-PAGE) in 15% polyacrylamide gels, with a stacking gel of 0.5% agarose (supplemented with bromophenol blue) for the insertion of the strip. The electrophoresis was performed using the Bio-Rad Mini-Protean Tetra system (Bio-Rad, Hercules, CA, USA) and the gels were submerged in 1X Tris-glycine running buffer at a constant voltage of 100 V for 80 min. Upon completion of the electrophoresis, the gels were stained with Sypro Ruby stain (Invitrogen™ Molecular Probes, Eugene, OR, USA) according to the manufacturer’s instructions. The analysis of the electrophoresis gels was performed using the Image Lab software version 6.1.0 (Bio-Rad Laboratories, Inc.), while the basic analysis of the 2D gels was performed using the PDQuest™ Basic 2-D Analysis software version 8.0.1 (Bio-Rad Laboratories, Inc., Hercules, CA, USA). Additionally, a second gel for each venom was electrotransferred to nitrocellulose membranes for subsequent evaluation by Western blot.

### 5.4. Western Blot of Sicarius thomisoides Venom with Anti-Phospholipase D Antibody

The different membranes with the transfer of the 2D electrophoresis of the venoms of *S. thomisoides* and *L. laeta* were used for the detection of phospholipase D proteins by Western blot using a polyclonal anti-phospholipase D1 antibody from *L. laeta* (anti-rLlPLD1) [25]. In this way, after electrophoresis, the gels were transferred to nitrocellulose membranes using the Trans-Blot Turbo system (Trans-Blot Turbo RTA Mini PVDF Transfer Kit, Bio-Rad, Hercules, CA, USA), and the quality of the transfer was evaluated by incubation with Ponceau S stain for one minute with agitation and the stain was removed by washing with distilled water. Subsequently, the membranes were blocked overnight with TBS-T-BSA blocking solution [Tris Buffered Saline with 0.1% Tween 20 (TBS-T) containing 2% BSA and 3% non-fat milk]. The blocked membrane was incubated for 1 h at room temperature with constant horizontal shaking with the anti-rLlPLD1 polyclonal mouse serum (dilution 1:1000). Once the incubation time had elapsed, the membranes were washed 6 times with TBS-T wash solution for 5 min each time and with horizontal agitation, and immediately incubated for 1 h at room temperature with agitation with the secondary antibody Goat anti-mouse IgG (H + L)-HRP (catalog number: 31430, Invitrogen™ Molecular Probes, Eugene, OR, USA) (dilution 1:40,000) in TBS-T. After the incubation and another 6 washes with TBS-T wash solution, the membranes were finally revealed using the SuperSignal™ WestFemto Maximum Sensitive Substrate kit (catalog Number: 34095, Thermo Fisher Scientific, Rockford, IL, USA) for subsequent analysis using the ChemiDoc™ Imaging System (catalog Number: 1708370, Bio-Rad Hercules, CA, USA).

### 5.5. RP-HPLC of Venoms and Fractioning of S. thomisoides Venom

An amount of 40 μg of venom from *S. thomisoides*, *L. laeta*, and the recombinant protein rLlPLD1 was prepared in 100 μL of sample buffer (chromatography-grade water, trifluoroacetic acid (TFA 0.1% and acetonitrile 5%) and submitted to reverse-phase high-performance liquid chromatography (RP-HPLC). The sample was deposited in the sample module of a Prominence LC-20A Modular HPLC system (Shimadzu Corporation, Nishinokyo Kuwabara-cho, Nakagyo-ku, Kyoto, Japan). Then, the chromatogram profile of each venom was obtained by separating the sample in a semi-preparative reverse phase column Viva C18 (250 × 4.6 mm), 5 μm, 300 Å (Catalog No. 9514575, Restek Corporation, Benner Circle Bellefonte, PA, USA), in a linear gradient with a mobile phase of 10 to 90% acetonitrile (ACN) in water + TFA 0.1% for a time of 60 min with an oven temperature of 25 °C and using a mobile phase flow of 0.5 mL/min. The absorbance peaks of the protein components of the venoms were obtained at a wavelength of 225 nm, which were graphed using LabSolutions software version 5.5.7 (Shimadzu Corporation, Nishinokyo Kuwabara-cho, Nakagyo-ku, Kyoto, Japan).

Once the chromatographic profile of *S. thomisoides* venom was identified, the peaks to be recovered were selected according to each retention time, and then a quantity of 100 μg of *S. thomisoides* venom in sample buffer was deposited in the sample receptacle of the SIL-20AC Autosampler module of the Shimadzu high-performance liquid chromatography system (Shimadzu Corporation, Nishinokyo Kuwabara-cho, Nakagyo-ku, Kyoto, Japan). Then, the sample was separated using a semi-preparative reverse-phase column Viva C18 (250 × 4.6 mm), 5 μm, 300 Å (Restek Corporation, Bellefonte, PA, USA), in a linear gradient with a mobile phase of 10 to 90% acetonitrile (ACN) in water + 0.1% TFA for a duration of 60 min with an oven temperature of 25 °C and using a mobile phase flow rate of 0.5 mL/min. Each sample fraction was collected according to the previously identified retention time range and recovered in 3.5 mL collection tubes (7 × 1 cm; Shimadzu), using the automated fractionation module FRC-10A Fraction Collector (Shimadzu Corporation, Nishinokyo Kuwabara-cho, Nakagyo-ku, Kyoto, Japan). Subsequently, the sample volume of each fraction was transferred to 1.5 mL tubes and dried under vacuum and centrifugation for 4–5 h at 37 °C in a SpeedVac Concentrator Plus (Eppendorf, Hamburg, Germany) to remove all solvents from the fractionation process. Finally, the dry samples were resuspended in 20 μL of 1X protein loading buffer for SDS-PAGE electrophoresis and subsequent Western blot or alternatively resuspended in 100 μL of 1X Amplex Red reaction buffer to evaluate the phospholipase D activity of each individual fraction.

### 5.6. Phospholipase D Activity of S. thomisoides Venom Fractions

The *S. thomisoides* venom fractions F1 to F16 were resuspended in 100 μL of 1X Amplex Red reaction buffer, and the phospholipase D activity of each obtained fraction was individually evaluated using the Amplex Red Sphingomyelinase-D Assay (Molecular Probes, Eugene, OR, USA) according to the manufacturer’s instructions and as previously documented [9]. In this way, the sample fractions F1 to F16, 20 μg/mL of *S. thomisoides* crude venom, and 20 μg/mL of *L. laeta* crude venom were dispensed in duplicate into a 96-well black flat-bottom microplate, and then 100 µL of the Amplex Red reaction mixture was added to each well. As a negative control, 1X reaction buffer was used, while as a positive control, H_2_O_2_ at a concentration of 10 µM was used. The assay was incubated at 37 °C for 1 h, and then the relative fluorescence was measured at an excitation of 530 nm and an emission of 590 nm in an Infinite M200 Pro fluorescence reader (Tecan Group Ltd., Männedorf, Switzerland). At least two assays were performed in duplicate for a statistical significance level of means with a *p*-value < 0.05.

### 5.7. Phospholipase D Detection from S. thomisoides Venom Fractions Using Western Blot

The fractions (F1 to F16) recovered from the venom of *S. thomisoides* were resuspended in 20 μL of 1X protein loading buffer, heated for 5 min at 95 °C, and then separated by SDS-PAGE electrophoresis on a 15% gel. Also, 5 μg of *S. thomisoides* venom and 2 μg of recombinant protein rLlPLD1 control, previously purified as documented [25], were used as positive detection control. The gel was then transferred to a nitrocellulose membrane, and the presence of proteins was confirmed by Ponceau S staining, followed by blocking with TBS-T-BSA blocking solution overnight with agitation, then incubated for 1 h at room temperature with horizontal agitation with a pool of polyclonal antibodies against recombinant PLD proteins from *L. laeta*, *L. intermedia*, *L. gaucho*, and *L. reclusa* (pAc anti-rPLDs) diluted 1:1000 in PBS-T buffer. Detection was performed with goat anti-mouse IgG (H+L) antibody conjugated with HRP at a dilution of 1:40,000 in PBS-T, followed by incubation with SuperSignal™ West Femto Maximum Sensitive Substrate reagent (Life Technologies, Carlsbad, CA, USA), and images were obtained using the ChemiDoc™ Imaging System (BIO-RAD, Hercules, CA, USA).

### 5.8. Sample Preparation of S. thomisoides Venom for Mass Spectrometry

An amount of 100 μg of total venom from *S. thomisoides* was precipitated by mixing the sample in a ratio of 1:8:1 with acetone and trichloroacetic acid (TCA) (sample:acetone:TCA) and then incubated overnight at −20 °C. Subsequently, the proteins were centrifuged at 14,000× *g* for 15 min and washed three times with cold acetone, then the solvent was discarded, and the precipitated proteins were dried by vacuum centrifugation in a SpeedVac Concentrator Plus (Eppendorf, Hamburg, Germany). Then, the precipitated protein extract was resuspended in 1X denaturing protein loading buffer and subjected to polyacrylamide gel electrophoresis (SDS PAGE), using a 4% stacking gel and a 10% separating gel, under electrophoretic conditions of 50 V for 20 min. Following this, the gel was stained with Coomassie blue, and the protein bands were excised and carefully cut into small pieces of 2 × 2 mm and deposited into 1.5 mL microcentrifuge tubes. Next, the staining was removed by adding 500 mL of 1:1 acetonitrile (ACN) and water, shaking the tubes in constant orbital agitation at 950 rpm for 10 min four times, and then removing the supernatant. Subsequently, the gel pieces were dehydrated by adding 500 mL of 100% ACN for 10 min with stirring, and the supernatant was removed before adding 500 mL of Tris-HCl (100 mM pH 8.0) solution and incubating with stirring for 15 min. Once the dehydration process was completed, the disulfide bonds of the cysteine residues in the proteins contained in the gel pieces were reduced using 10 mM dithiothreitol for 30 min at 56 °C, and then the thiol groups were alkylated by adding 10 mM iodoacetamide and incubating for 30 min in the dark at room temperature. Next, the gel pieces were dehydrated by removing the liquid using 100% ACN. Subsequently, the ACN was removed by pipetting, and then the pieces were dried in an Eppendorf SpeedVac Concentrator Plus (Eppendorf SE, Hamburg, Germany). The pieces were rehydrated in 100 mM Tris-HCl pH 8 with 10 mM CaCl_2_ containing 60 ng/mL of trypsin (Promega, Madison, WI, USA) in a 5:1 protein:trypsin ratio. The tubes were kept on ice for 1 h and incubated for 16 h at 37 °C. The digestion was stopped by adding 10 mL of 20% formic acid (FA) to the supernatant containing the peptides at a pH < 3. Finally, the recovered supernatant was dried in an Eppendorf SpeedVac Concentrator Plus for 4 h, and the peptides were finally desalted using OMIX Pippete Tips C18 (Agilent Technologies, Santa Clara, CA, USA). The processed venom sample of *S. thomisoides* was analyzed using mass spectrometry.

### 5.9. Reverse-Phase Liquid Chromatography–Tandem Mass Spectrometry (LC-MS/MS) and Sequence Analysis of Proteins from the Venom of Sicarius thomisoides

The mass spectrometry analysis of *S. thomisoides* spider venom was conducted in collaboration and in the laboratory of Dr. Leonard Foster at the University of British Columbia (Vancouver Point Grey Campus 436–2125, East Mall Vancouver, Vancouver, BC, Canada) using the following protocol. The concentration of peptides was measured using NanoDrop One (ThermoFisher, Waltham, MA, USA, A205, scopes), and 50 ng of peptides were loaded into TimsTOF Pro2 (Bruker Daltonics, Billerica, MA, USA), using a CaptiveSpray source coupled to nanoElute UHPLC (Bruker Daltonics, Billerica, MA, USA) with a 25 cm Bruker’s PepSep™ ULTRA C18 HPLC column (25 cm × 75 μm inner diameter, generating particle sizes of 1.9 μm with 20 μm emitter), and then heated to 50 °C. Next, a standard 30 min gradient was used, consisting of buffer A and B, where buffer A contained 0.1% formic acid (FA) and 0.5% ACN in water, and buffer B consisted of 0.1% FA and 99.4% ACN in water. The percentage of buffer B in the standard gradient increased from 2% to 12% over 15 min and ended with 33% after 15 min. The column was washed with 95% buffer B for over 8 min. The temperature of the NanoElute thermostat was maintained at 7 °C, and the analysis was performed with a mobile phase flow rate of 0.4 μL/min.

Subsequently, the TimsTOF Pro2 system was programmed using the Data Independent Acquisition (DIA) method by Neural Networks (NN), in DIA PASEF mode with positive polarity for the MS scan window from 100 to 1700 *m*/*z*. The capillary voltage was set to 1700 V, with gasification of 3 L/min and a drying temperature of 200 °C. The MS spectra were collected from *m*/*z* 100 Th to *m*/*z* 1700 Th and from the ion mobility range (1/K0) 0.7 V·s/cm^2^ to 1.3 V·s/cm^2^. The TIMS was operated with equal ramp and accumulation times of 100 ms (100% duty cycle). For each TIMS cycle, seven dia-pasef scans were used, each with 3–4 steps, with a total of 25 dia-pasef windows spanning from *m*/*z* 299.5 Th to *m*/*z* 1200.5 Th and from ion mobility range (1/K0) 0.7 to 1.3 V·s/cm^2^. Variable isolation width from *m*/*z* 36 Th- *m*/*z* 61 Th was used with an overlap of *m*/*z* 1 Th between two neighboring windows. The collision energy was ramped linearly stepped as a function of mobility value from 20 eV at 1/K0 = 0.6 V·s/cm^2^ to 65 eV at 1/K0 = 1.6 V·s/cm^2^.

Data acquisition was performed using DIA-NN (PMID31768060) software version 1.9.1, with a library-independent search method, using a combination of protein sequences from *L. laeta* and *S. thomisoides* available in the UniProt (https://www.uniprot.org/) and NCBI (https://www.ncbi.nlm.nih.gov/) databases, and manually curated using 226 entries for common contaminants. Other search parameters include trypsin/P digestion mode with 1 missed cleavage, 1 maximum number of variable options enabled, peptide length ranging from 7 to 30, precursor charge ranging from 2 to 4, precursor and fragment ion *m*/*z* ranging from 200 to 1700. Precursor FDR was set to 1%, with 0 mass accuracy and MS1 accuracy (for “auto” option of mass tolerance), enabling heuristic protein inference, use of isotopologues, match between run (MBR), and no shared spectra. Protein inference is set as “Protein name from FASTA”, Quantification Strategy, RT-dependent mode for Cross-run normalization, and IDs, RT and IM Profiling mode for library generation. The identification of the proteins was carried out using different databases for peptides in FASTA format from the taxa of the Sicariidae family (6919), *Sicarius* (571535) and *Loxosceles* (6920), available at UniProt (https://www.uniprot.org/), as well as sequences for *S. thomisoides* (43 sequences) and *L. laeta* (57 sequences) available at NCBI (https://www.ncbi.nlm.nih.gov/) using Blastp, to subsequently be grouped according to sequence type into homology groups, and the type of protein and function was identified.

The mass spectrometry proteomics data have been deposited to the ProteomeXchange Consortium via the PRIDE (Pubmed ID: 34723319) partner repository with the data set identifier PXD062481 “Proteome of the six-eyed sand-spider *Sicarius thomisoides*”.

## Figures and Tables

**Table 1:** Proteins of *Sicarius thomisoides* venom identified by liquid chromatography–tandem MS/MS.

Category	Top Protein ID Sequence Match	Description/Function	N° Total Unique Sequences Detected	Predicted MW (kDa) *	Organism
Toxins	A0A0D4WV12	Dermonecrotic toxin StSicTox-betaIB1i	9	31.8	*Sicarius terrosus*
	C0JB33	Dermonecrotic toxin SpeSicTox-betaIB3 (Fragment)	7	34.3	*Sicarius peruensis*
	C0JB34	Dermonecrotic toxin SpeSicTox-betaIB4 (Fragment)	1	31.8	*Sicarius peruensis*
	C0JB52	Dermonecrotic toxin SpaSicTox-betaIF1 (Fragment)	3	30.4	*Sicarius patagonicus*
	C0JB53	Dermonecrotic toxin SpeSicTox-betaIF1 (Fragment)	4	31.3	*Sicarius peruensis*
	C0JB54	Dermonecrotic toxin StSicTox-betaIF1 (Fragment)	2	32.1	*Sicarius terrosus*
	C0JB55	Dermonecrotic toxin SdSicTox-betaIF1 (Fragment)	3	31.8	*Sicarius damarensis*
	C0JB56	Dermonecrotic toxin SpeSicTox-betaIIA3i (Fragment)	2	31.7	*Sicarius peruensis*
	C0JB62	Dermonecrotic toxin SpeSicTox-betaIIA2iv (Fragment)	8	31.8	*Sicarius peruensis*
	C0JB65	Dermonecrotic toxin SpeSicTox-betaIIA2v (Fragment)	1	31.8	*Sicarius peruensis*
	C0JB70	Dermonecrotic toxin SpaSicTox-betaIIA3 (Fragment)	1	31.7	*Sicarius patagonicus*
	C0JB93	Dermonecrotic toxin LspiSicTox-betaIII2 (Fragment)	2	31.6	*Loxosceles spinulosa*
	C0JB94	Dermonecrotic toxin LspiSicTox-betaIII1 (Fragment)	1	31.9	*Loxosceles spinulosa*
	C0JB40	Dermonecrotic toxin LcsSicTox-betaIC1 (Fragment)	4	31.8	*Loxosceles spinulosa*
	C0JB41	Dermonecrotic toxin LspiSicTox-betaIE2i (Fragment)	1	31.2	*Loxosceles spinulosa*
	C0JB43	Dermonecrotic toxin LspiSicTox-betaIE2iii (Fragment)	2	31.2	*Loxosceles spinulosa*
	C0JB46	Dermonecrotic toxin LspiSicTox-betaIE4i (Fragment)	2	31.4	*Loxosceles spinulosa*
	A0A0D4WTV1	Dermonecrotic toxin LarSicTox-betaID1 (Fragment)	2	33.1	*Loxosceles arizonica*
	C0JB09	Dermonecrotic toxin LarSicTox-alphaIII1 (Fragment)	1	31.5	*Loxosceles arizonica*
	C0JB30	Dermonecrotic toxin LarSicTox-alphaVII1 (Fragment)	2	31.6	*Loxosceles arizonica*
	C0JB07	Dermonecrotic toxin LapSicTox-alphaII1 (Fragment)	1	31.2	*Loxosceles apachea*
	C0JB06	Dermonecrotic toxin LvSicTox-alphaII1 (Fragment)	3	31.5	*Loxosceles variegata*
	C0JB14	Dermonecrotic toxin LhSicTox-alphaIV1i (Fragment)	1	31.3	*Loxosceles hirsuta*
	A0A0E3STQ7	Sphingomyelinase D-like protein (Fragment)	2	31.1	*Loxosceles* sp
	A0A0E3SV20	Sphingomyelinase D-like protein (Fragment)	1	31.6	*Loxosceles rufescens*
	A0A1B2AS99	Loxtox protein	2	34.5	*Loxosceles similis*
	A0A1B2ASA6	Loxtox protein (Fragment)	1	30.7	*Loxosceles similis*
	A0A1B2ASA7	Loxtox protein	3	38.6	*Loxosceles similis*
	A0A1B2ASA9	Loxtox protein (Fragment)	2	20.9	*Loxosceles similis*
	A0A1B2ASB2	Loxtox protein	2	34.9	*Loxosceles similis*
	A0A1B2ASC7	Loxtox protein	5	34.8	*Loxosceles similis*
	A0A1B2ASF4	Loxtox protein	3	35.5	*Loxosceles similis*
	A0A1B2ASE8	PLD-Ls protein	1	54.3	*Loxosceles similis*
	A0A6B9KJ60	Membrane metalloendopeptidase (Fragment)	2	34.5	*Loxosceles reclusa*
	A0A6B9KRY4	Membrane metalloendopeptidase (Fragment)	3	59.6	*Loxosceles spinulosa*
	A0FKN6	Astacin-like metalloprotease toxin 1	5	30.4	*Loxosceles intermedia*
	A0A6B9KDY5	Neuroendocrine convertase S8 peptidase (Fragment)	5	67.3	*Loxosceles rufescens*
	A0A6B9KDZ4	Venom gland peptide U17-PHTX-Pmx1c	2	9.9	*Loxosceles reclusa*
	A0A6B9KL70	Venom allergen/CRISP (Fragment)	2	46.4	*Loxosceles reclusa*
Non-toxins	A0A343ZHI3	Histone 3 (Fragment)	4	-	*Loxosceles rufescens*
	A0A6B9KE20	Actin	18	-	*Loxosceles arizonica*
	A0A6B9KL18	Tropomyosin (Fragment)	3	-	*Loxosceles rufescens*
	A0A023J1U5	NADH dehydrogenase subunit 1 (Fragment)	1	-	*Loxosceles* sp
	A0A023J2D9	NADH dehydrogenase subunit 1 (Fragment)	1	-	*Loxosceles* sp
	A0A023J4P5	NADH dehydrogenase subunit 1 (Fragment)	1	-	*Loxosceles rufescens*
	B8R316	NADH-ubiquinone oxidoreductase chain 1 (Fragment)	1	-	*Loxosceles* sp
	A0A4P8VXV3	Cytochrome b	1	-	*Loxosceles similis*
	A0A650CBZ5	Cytochrome c oxidase subunit 1 (Fragment)	1	-	*Loxosceles* sp
	A0A097HWB9	Cytochrome c oxidase subunit 1 (Fragment)	1	-	*Loxosceles rufescens*
	C1ITM5	Cytochrome c oxidase subunit 1 (Fragment)	1	-	*Loxosceles intermedia*
	C1ITN2	Cytochrome c oxidase subunit 1 (Fragment)	1	-	*Loxosceles spinulosa*
	C1ITN6	Cytochrome c oxidase subunit 1 (Fragment)	1	-	*Loxosceles spinulosa*
	C1ITP4	Cytochrome c oxidase subunit 1 (Fragment)	1	-	*Sicarius rupestris*
	C1ITP9	Cytochrome c oxidase subunit 1 (Fragment)	1	-	*Sicarius damarensis*
	C1ITQ3	Cytochrome c oxidase subunit 1 (Fragment)	1	-	*Sicarius terrosus*
	C1ITQ5	Cytochrome c oxidase subunit 1 (Fragment)	1	-	*Sicarius rugosus*

* Based on molecular mass from homologous protein match.

## Data Availability

The original contributions presented in this study are included in the article. Further inquiries can be directed to the corresponding author.
